# Natural Killer Cells Integrate Signals Received from Tumour Interactions and IL2 to Induce Robust and Prolonged Anti-Tumour and Metabolic Responses

**DOI:** 10.20900/immunometab20190014

**Published:** 2019-09-25

**Authors:** Nidhi Kedia-Mehta, Chloe Choi, Aisling McCrudden, Elisabeth Littwitz-Salomon, Proinnsias G. Fox, Clair M. Gardiner, David K. Finlay

**Affiliations:** 1 School of Biochemistry and Immunology, Trinity Biomedical Sciences Institute, Trinity College Dublin, 152-160 Pearse Street, Dublin 2, Ireland; 2 School of Pharmacy and Pharmaceutical Sciences, Trinity Biomedical Sciences Institute, Trinity College Dublin, 152-160 Pearse Street, Dublin 2, Ireland

**Keywords:** natural killer cell, metabolism, NK1.1, tumour, cancer, receptor, IL2, CD25, mTORC1, cMyc, glycolysis, OXPHOS, Slc7a5

## Abstract

Natural Killer (NK) cells are lymphocytes with an important role in anti-tumour responses. NK cells bridge the innate and adaptive arms of the immune system; they are primed for immediate anti-tumour function but can also have prolonged actions alongside the adaptive T cell response. However, the key signals and cellular processes that are required for extended NK cell responses are not fully known. Herein we show that murine NK cell interaction with tumour cells induces the expression of CD25, the high affinity IL2 receptor, rendering these NK cells highly sensitive to the T cell-derived cytokine IL2. In response to IL2, CD25^high^ NK cells show robust increases in metabolic signalling pathways (mTORC1, cMyc), nutrient transporter expression (CD71, CD98), cellular growth and in NK cell effector functions (IFNγ, granzyme B). Specific ligation of an individual activating NK cell receptor, NK1.1, showed similar increases in CD25 expression and IL2-induced responses. NK cell receptor ligation and IL2 collaborate to induce mTORC1/cMyc signalling leading to high rates of glycolysis and oxidative phosphorylation (OXPHOS) and prolonged NK cell survival. Disrupting mTORC1 and cMyc signalling in CD25^high^ tumour interacting NK cells prevents IL2-induced cell growth and function and compromises NK cell viability. This study reveals that tumour cell interactions and T cell-derived IL2 cooperate to promote robust and prolonged NK cell anti-tumour metabolic responses.

## Introduction

Natural Killer (NK) cells play an important role in our immune response against cancer. They can recognise transformed tumour cells and directly kill them through various mechanisms including the directed release of cytotoxic granules. NK cells also produce the cytokine Interferon γ (IFNγ) that can directly kill tumour cells as well as serving to communicate to other immune cells, thus orchestrating the immune response. As part of the innate immune system NK cells have an inherent ability to kill target cells without prior sensitisation; NK cells have a range of receptors that can distinguish between normal cells and transformed cells. Additionally, the cytotoxic activity of NK cells can be further induced by signals including cytokines such as the adaptive cytokine Interleukin 2 (IL2), which is primarily released by activated T cells [1–3]. Therefore, NK cells have an important role in the anti-tumour response that bridges the innate and adaptive arms of the immune system.

Recent discoveries have identified cellular metabolism as an integral factor in the control of cytokine-induced NK cells responses. Any perturbation that impacts upon NK cell metabolism impairs the effector function of these cytokine-stimulated NK cells including IFNγ production and tumour cell cytotoxicity [4–9]. Cytokine-induced NK cell metabolism and effector responses have also been shown to be dysfunctional in the context of diseases such as obesity and cancer [10–12]. The immediate capacity of NK cells to produce IFNγ and kill target cells in response to activating receptor ligation is not associated with increased cellular metabolism [13]. However, it is not clear whether prolonged NK cell responses to tumour cells and activating ligands require increased metabolism in order to support NK cells’ energy and biosynthetic demands. Also, while it is known that NK cells can operate alongside antitumour T cells responses [14,15], how the signals received from tumour cell interactions (NK receptor ligands) and T cell derived signals (IL2) cooperate with respect to NK cell metabolic and functional responses is not known.

This study has investigated how tumour interactions and the ligation of the activating NK cell receptor NK1.1 impact upon NK cell metabolism and NK cell effector outputs. NK cell interactions with tumour cells or specific NK cell receptor ligation results in the expression of the high affinity IL2 receptor CD25. These induced CD25^high^ NK cells can respond to IL2, leading to increased NK cell metabolism, function and survival. This study shows how innate and adaptive stimuli delivered through target cell interactions and the T cell-derived cytokine IL2, respectively, integrate to promote robust and prolonged NK cell metabolic and functional responses.

## Results

### CD25 Expression on Tumour-Interacting NK Cells Is Required for Increased Metabolism in Response to IL2 Cytokine

**Figure 1:**
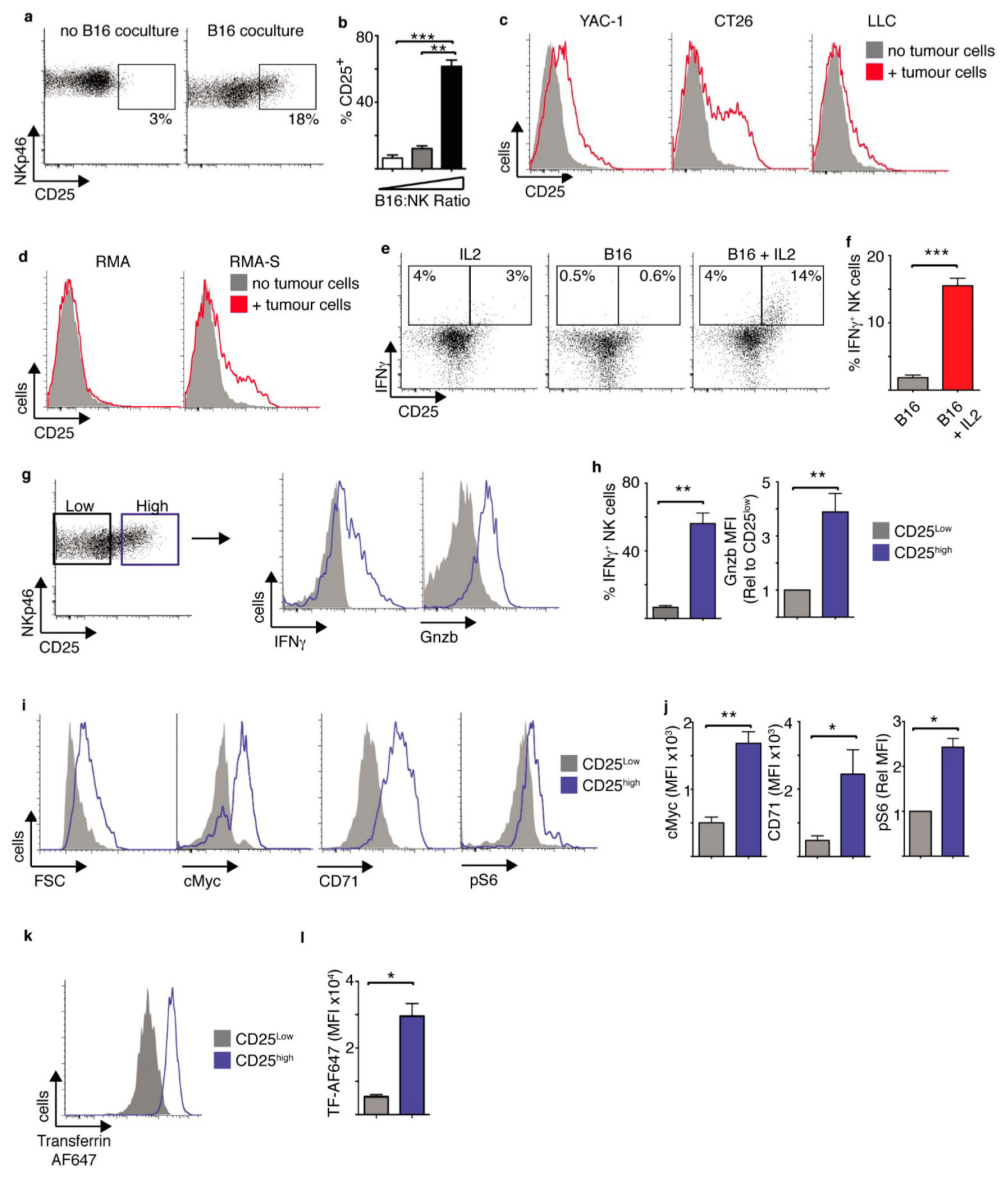
Tumour interactions induce CD25^high^ NK cells with heightened metabolism and effector function. (**a–d**) Cultured NK cells (6 days in IL15 10 ng/mL) were purified and then co-cultured with or without B16 melanoma cells at an E:T ratio of 2:1 (**a**), with B16 melanoma cells at an E:T ratio of 4:1, 2:1 or 1:2 (**b**), with or without YAC-1, CT26 and LLC tumour cells at an E:T ratio of 1:4 (**c**), or with or without RMA/RMA-S cells at an E:T ratio of 1:4 (**d**) for 18 h before analysis of CD25 expression by flow cytometry. (**e**–**k**) Cultured NK cells (6 days in IL15 10 ng/mL) were purified and then co-cultured with B16 melanoma cells for 18 h at an E:T ratio of 2:1, washed and put back into culture with IL15 (7.5 ng/mL) with or without ± IL2 (20 ng/mL) for 18 h before analysis by flow cytometry. (**e**) IFNγ production by CD25^high^ and CD25^neg^ NK cells cultured in IL2 alone, with B16 cells or with B16 cells plus IL2. (**f**) IFNγ production in CD25^high^ NK cells cultured with B16 ± IL2. (**g**,**h**) Analysis of NK cells cultured with B16 cells + IL2 comparing CD25^high^ and CD25^neg^ NK cells (g, left panel) for effector functions, IFNγ production and granzyme (Gnzb) B expression. (**i**,**j**) Analysis of cell size (FSC), cMyc and CD71 expression, and levels of phosphorylated S6 ribosomal protein (pS6) in CD25^high^ and CD25^neg^ NK cells from B16 cells + IL2 co-cultures. (**k,l**) Rates of fluorescent transferrin uptake were measured in CD25^high^ and CD25^neg^ NK cells from B16 cells + IL2 co-cultures. Data is representative (**a**,**c**,**d**,**e**,**g**,**i**,**k**) or mean ± SEM **(b,f,h,j,l)** of 3–5 independent experiments. Data was analyzed using a paired students *t*-test. (* *p* < 0.05, ** *p* < 0.01, *** *p* < 0.001).

NK cells and T cells are both important for anti-tumour responses. To understand how stimuli from tumour cells and IL2 from local T cells might affect NK cells responses, we explored NK cell responses when co-cultured with tumour cells in the presence or absence of exogenously supplied IL2. During initial experiments splenic NK cells were cultured for 6 days in low dose IL15, a cytokine required for Dendritic cell-mediated NK cell priming *in vivo* [16–18] (called “cultured NK cells” hereafter), purified by magnetic bead cell sorting prior to being co-cultured with B16 melanoma cells for 18 h. Interactions with B16 tumour cells resulted in the expression of high levels of CD25, the high affinity IL2 receptor subunit, on a proportion of NK cells (Figure 1a,b). Increased CD25 expression was also observed when NK cells were cultured with other murine tumour cells including YAC-1 cells (T cell lymphoma) CT26 cells (colon carcinoma) and LLC cells (Lewis Lung carcinoma) cells, though to differing degrees (Figure 1c, Supplementary Figure S1a). Similarly, culturing NK cells with RMA lymphoma cells that are sensitive to NK cells killing (RMA-S cells) resulted in CD25 expression on the NK cells. In contrast, culturing NK cells with RMA lymphoma cells that are insensitive to NK cells killing (parental RMA cells) did not (Figure 1d, Supplementary Figure S1b). While CD25 is often considered a marker of activated T cells, this is not always the case for NK cells. For instance, NK cells are robustly activated by high dose IL-15 (100 ng/mL) but this cytokine does not induce the expression on CD25 (Supplementary Figure S1c). In fact, in terms of cytokines that activate NK cells, it is primarily IL12 that induces the expression of CD25 expression in murine NK cells even though IL12 does not potently activate NK cells alone [1,5].

We next explored how the expression of CD25 affected the way NK cells responded to the T cell cytokine IL2. Following 18 h co-culture with B16 cells, IL2 was added for a further 18 h and the NK cells initially analysed for IFNγ production. It was clear that the CD25^high^ expressing NK cells, but not the CD25_low_ NK cells, produced IFNγ in the presence of IL2 (Figure 1e,f). Tumour interacting NK cells did not produce IFNγ in the absence of IL2 (Figure 1e,f). Similarly, NK cells cultured in the absence of tumour cells for 18 h and then provided IL2 for 18 h did not produce IFNγ (Figure 1e). These CD25^high^ NK cells were not a particular subset; all three of the main NK cell subsets were present (CD27^+^ CD11b^−^, CD27^+^ CD11b^+^, CD11b^+^ CD27^−^) (Supplementary Figure S1d). To further understand the impact of CD25 expression and IL2 on NK cell metabolism and function, CD25^high^ and CD25_low_ NK cells from B16-NK cell co-cultures were compared (Figure 1g, left panel). In the presence of IL2, CD25^high^ NK cells produced large amounts of IFNγ and had substantially higher expression of the cytotoxic granule component granzyme B compared to CD25_low_ NK cells (Figure 1g,h). CD25^high^ NK cells were also larger than CD25_low_ NK cells, suggesting increased cellular growth (Figure 1i). Indeed, there was increased expression of the metabolic regulator cMyc and increased expression of the cMyc target gene CD71, the transferrin receptor, in CD25^high^ NK cells compared to the CD25_low_ counterparts (Figure 1i,j). mTORC1 is another metabolic regulator that is known to be important in the regulation of NK cell metabolism and function [5,19]. The levels of mTORC1 signalling, measured by the phosphorylation of S6 ribosomal protein, were also elevated in CD25^high^ NK cells compared to CD25_low_ NK cells (Figure 1i,j). However, there was not a general increase in protein expression on CD25^high^ NK cell; CD25_low_ and CD25^high^ NK cells expressed equivalent levels of the NK cell receptors NK1.1 and NKp46 (Supplementary Figure S1e). To confirm that the CD25^high^ NK cells have increased levels of metabolism we used a metabolic uptake assay looking at the amount of fluorescently labelled transferrin taken up by these NK cells. CD25^high^ NK cells had substantially increased levels of transferrin compared to CD25_low_ NK cells (Figure 1j,k).

**Figure 2:**
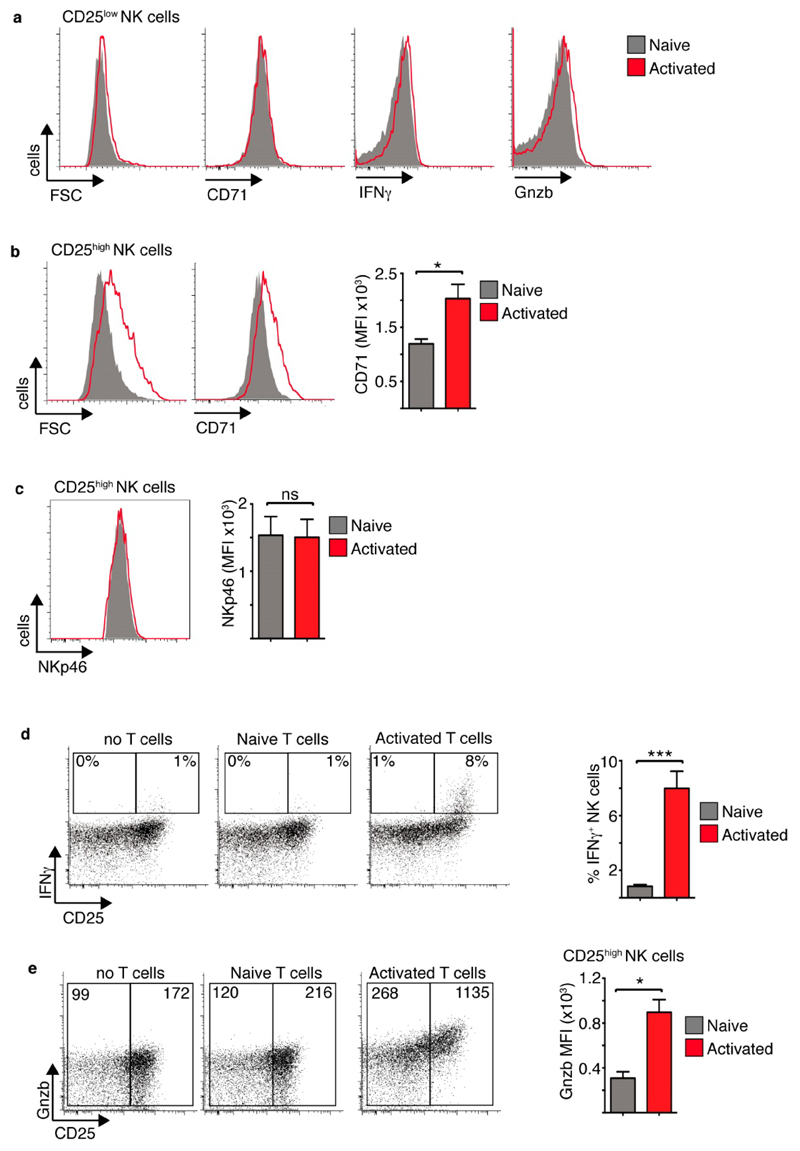
Activated T cells support increased metabolism and function of CD25^high^ tumour interacting NK cells. Cultured NK cells (6 days in IL15 10 ng/mL) were purified and then co-cultured with B16 melanoma cells and purified T cells at NK:T:B16 ratio of 1:1:2. The T cells were either naïve or αCD3/αCD28-activated T cells. The NK cells were analysed by flow cytometry for cell size and CD71 (**a**,**b**), NKp46 expression (**c**) IFNγ production and Gnzb expression (**a**,**d**,**e**) in CD25^low^ and CD25^high^ NK cells as indicated. Data is representative (**a**–**e**) or mean ± SEM (**b**–**e**) of 4 independent experiments. Data was analyzed using a paired students *t*-test. (* *p* < 0.05, *** *p* < 0.001, ns non-significant).

These results suggested that tumour interactions would allow NK cells to respond to IL2 produced locally by T cells. To test this premise NK cells were co-cultured with B16 tumour cells and also either naïve T cells or T cells activated with anti-CD3 and anti-CD28 (Supplementary Figure S2). T cells were added at a 1:1 ratio to the NK cells. CD25_low_ NK cells behaved equivalently in the presence of naïve or activated T cells (Figure 2a). In contrast, CD25^high^ NK cells were larger and expressed increased levels CD71 in the presence of activated T cells compared to naïve T cells (Figure 2b). The expression of the surface receptor NKp46 was unchanged (Figure 2c). Additionally, CD25^high^ NK cells cultured with activated T cells produced more IFNγ and expressed higher levels of granzyme B compared CD25^high^ NK cells cultured with naïve T cells or no T cells (Figure 2d,e). The fact that only CD25^high^ NK cells showed evidence of enhanced metabolism and function suggested that the factor produced by the activated T cells, but not naïve T cells, to mediate these effects is the cytokine IL2.

Taken together, these data support a model whereby NK cell-tumour interactions facilitated the upregulation of CD25 expression allowing NK cells to respond to T cell-derived IL2, which induced metabolic signalling pathways, cellular growth and robust NK cell effector function.

### NK1.1 Receptor Ligation Facilitates IL2-Dependent Metabolic Reprogramming

**Figure 3:**
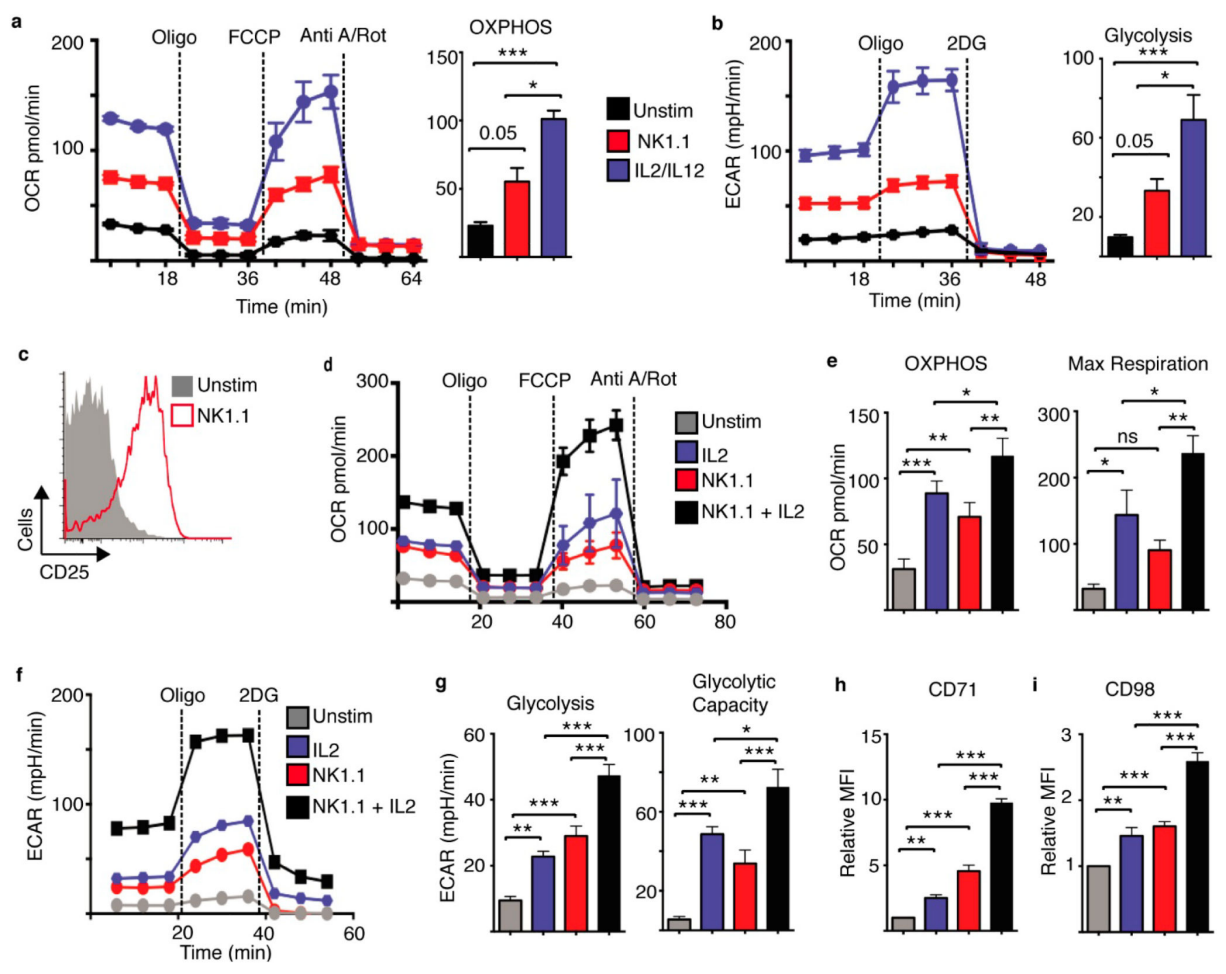
NK1.1 activating receptor ligation facilitates IL2-mediated metabolic responses in NK cells. Cultured NK cells (6 days in IL15 10 ng/mL) were purified and stimulated with a plate bound α-NK1.1 antibody (10 μg/mL) in media supplemented with low dose IL15 (5 ng/mL) ± IL2 (20 ng/mL) as indicated, with IL2 (20 ng/mL) plus IL12 (10 ng/mL) or left unstimulated (IL15 - 5 ng/mL). Cells were analysed for rates of glycolysis (**a,f,g**) and OXPHOS (**b,d,e**) using the seahorse extracellular flux analyser or by flow cytometry for the expression of CD25 (**c**), CD71 (**h**) or CD98 (**i**). Data is representative (**a–d,f**) or mean ± SEM **(a,b,e, g–i**) of 4–5 independent experiments. Data was analyzed using a one way ANOVA and tukey post test (* *p* < 0.05, ** *p* < 0.01, *** *p* < 0.001, ns non-significant).

To further study these metabolic responses we focused on a model system and investigated how the archetypal NK cell activating receptor, NK1.1, integrates with IL2 signalling to regulate NK cell metabolic pathways and promote robust effector responses. Previous research showed that short-term ligation of the NK1.1 activating receptor induces IFNγ production from NK cells without engaging any changes in cellular metabolism [13]. Here we investigated NK cell metabolism following activation of the NK1.1 receptor for 18 h in comparison to NK cells stimulated with IL2 plus IL12 cytokine. As described previously, IL2/IL12 stimulated NK cells engaged a robust metabolic response with increased rates of OXPHOS and maximal respiratory capacity (Figure 3a)[5]. NK1.1 stimulated NK cells also showed increased rates of OXPHOS, though these rates were significantly less than those of IL2/IL12 stimulated NK cells (Figure 3a). Similarly, while both IL2/IL12 and NK1.1 stimulated NK cells had increased rates of glycolysis and glycolytic capacity, metabolic rates were significantly less in NK1.1-stimulated NK cells compared to IL2/IL12 stimulated NK cells (Figure 3b).

As was observed for NK cells co-cultured with B16 tumour cells, NK1.1 stimulated NK cells increased the expression of CD25, suggesting that these cells would be more responsive to IL2 cytokine (Figure 3c). Indeed, NK1.1 plus IL2-stimulated NK cells had substantially high rates of glycolysis and OXPHOS compared to NK cells stimulated with either IL2 or NK1.1 alone (Figure 3d–g). NK cells stimulated with NK1.1 plus IL2 also had the highest expression of nutrient transporters including the transferrin receptor, CD71, and CD98, a component of the system L amino acid transporter (Figure 3h,i, Supplementary Figure S3). Therefore, NK1.1 sensitises NK cells for robust IL2-dependent metabolic responses.

**Figure 4:**
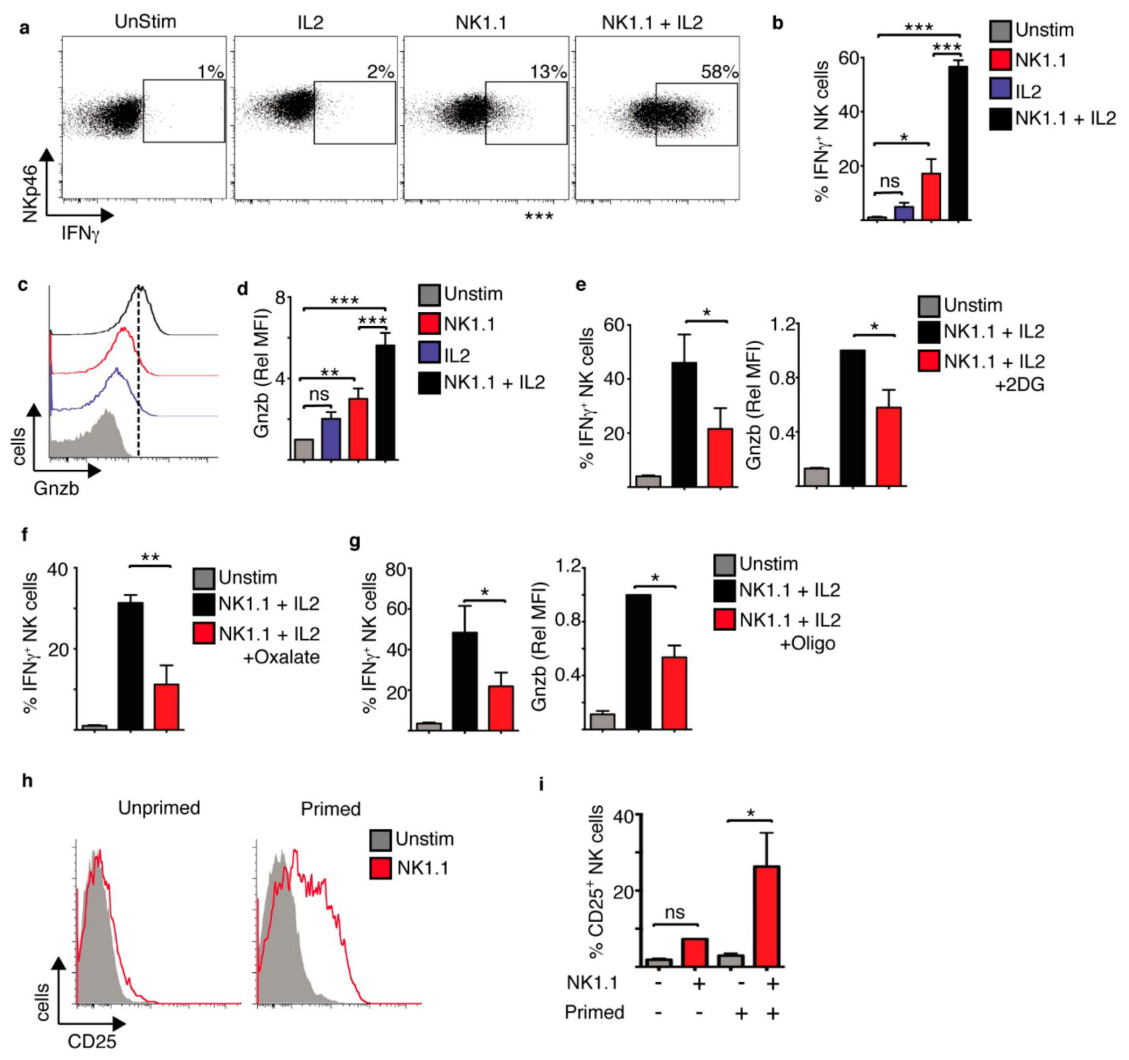
NK1.1 activating receptor ligation plus IL2 facilitates robust NK cell effector function. Cultured NK cells (6 days in IL15 10 ng/mL) were stimulated with a plate bound α-NK1.1 antibody (10 μg/mL) in media supplemented with low dose IL15 (5 ng/mL) ± IL2 (20 ng/mL) as indicated, or left unstimulated (IL15; 5 ng/mL). Inhibitors of glycolysis, 2-deoxyglucose (2DG, 1 mM) or oxalate (2 mM), or OXPHOS, oligomycin (4 nM) were added as indicated. Cells were analysed by flow cytometry for the production of IFNγ (**a,b,e–g**) and the expression of granzyme B (**c-e,g**). (**h,i**) NK cells were purified directly *ex vivo* from murine splenocytes and stimulated with plate bound α-NK1.1 antibody (unprimed). Alternatively splenocytes were maintained for 18 h in IL15 (10 ng/mL) prior to NK cell purification and stimulation with plate bound α-NK1.1 antibody (primed). Cells were analysed for CD25 expression by flow cytometry. Data is representative (**a,c,h**) or mean ± SEM (**b,d–g,i**) of 3–5 independent experiments. Data was analyzed using a one way ANOVA and tukey post test (* *p* < 0.05, ** *p* < 0.01, *** *p* < 0.001, ns non-significant).

These heightened metabolic responses in NK1.1 plus IL2 stimulated NK cells paralleled NK cells functional responses. NK cells stimulated with IL2 alone did not produce IFNγ, while a small proportion of NK cells stimulated with NK1.1 alone produced a small amount of IFNγ. In contrast, the majority of NK cells stimulated with NK1.1 plus IL2 produced high levels of IFNγ cytokine (Figure 4a,b), as seen in a previous study [20]. Similarly, while there was increased expression of granzyme B in NK cells stimulated with NK1.1 alone, NK cells stimulated with NK1.1 plus IL2 had significantly higher expression of this cytotoxic protein (Figure 4c,d). Heightened cellular metabolism was essential for these elevated NK cell effector function as limiting the flux through glycolysis using the glycolytic inhibitors 2-deoxyglucose or oxalate, and limiting OXPHOS using nanomolar concentrations of oligomycin, impaired NK cell IFNγ production and granzyme B expression (Figure 4e–g, Supplementary Figure S4).

As experiments were performed on NK cell primed with low dose IL15 cytokine we considered whether priming is important for NK cell receptor-induced expression of CD25. NK cells were purified from splenocytes directly *ex vivo* and stimulated with anti-NK1.1 (unprimed) or alternatively NK cells were primed for 24 h with low dose IL15 (10 ng/mL) before purification and stimulation. NK1.1 stimulation induced a substantial increase in CD25 expression in primed but not unprimed splenic NK cells (Figure 4h). Therefore, as with other aspects of NK cell biology, the data demonstrates that priming of NK cells is required for NK cell receptor-mediated induction of CD25 expression.

### mTORC1 and cMyc Are Required for NK1.1 Plus IL2-Induced Responses

**Figure 5:**
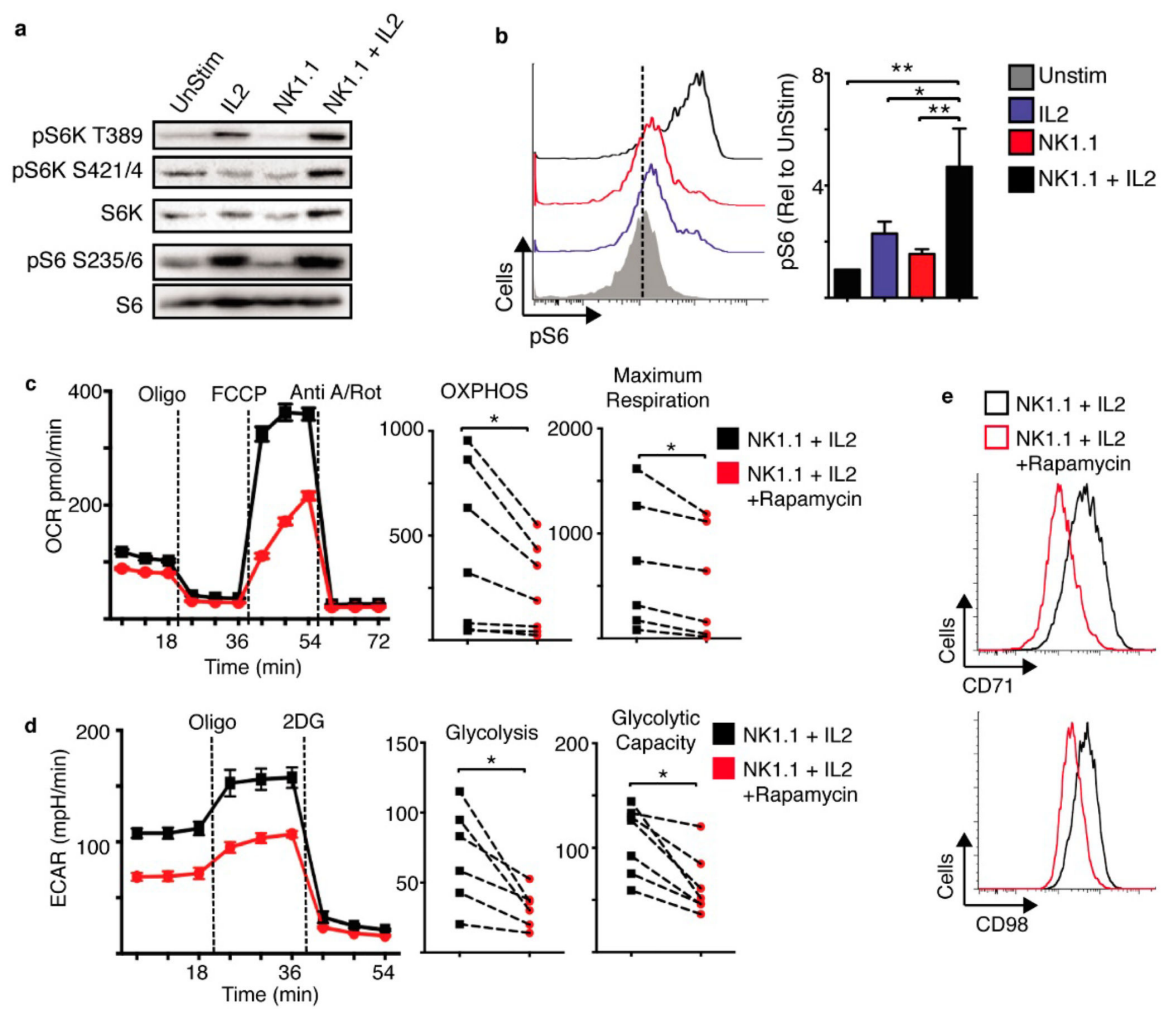
mTORC1 is required for NK1.1 plus IL2-induced NK cell metabolism. (**a–e**) Cultured NK cells (6 days in IL15 10 ng/mL) were purified and stimulated with a plate bound α-NK1.1 antibody (10 μg/mL) in media supplemented with low dose IL15 (5 ng/mL) ± IL2 (20 ng/mL) ± rapamycin (20 nM) as indicated, or left unstimulated (IL15; 5 ng/mL). Cells were analysed by immunoblot analysis (**a**), by flow cytometry for levels of phosphorylated S6 ribosomal protein (pS6) (**b**), or the expression of CD71 and CD98 (**e**). Alternatively, rates of OXPHOS (**c**) or glycolysis (d) were measured using the seahorse extracellular flux analyser. Data is representative (**a–e**) or mean ± SEM (**b–d**) of 5–6 independent experiments. Data was analyzed using a one way ANOVA and tukey post test (**b**), a paired students *t*-test (**c,d**) (* *p* < 0.05, ** *p* < 0.01).

**Figure 6:**
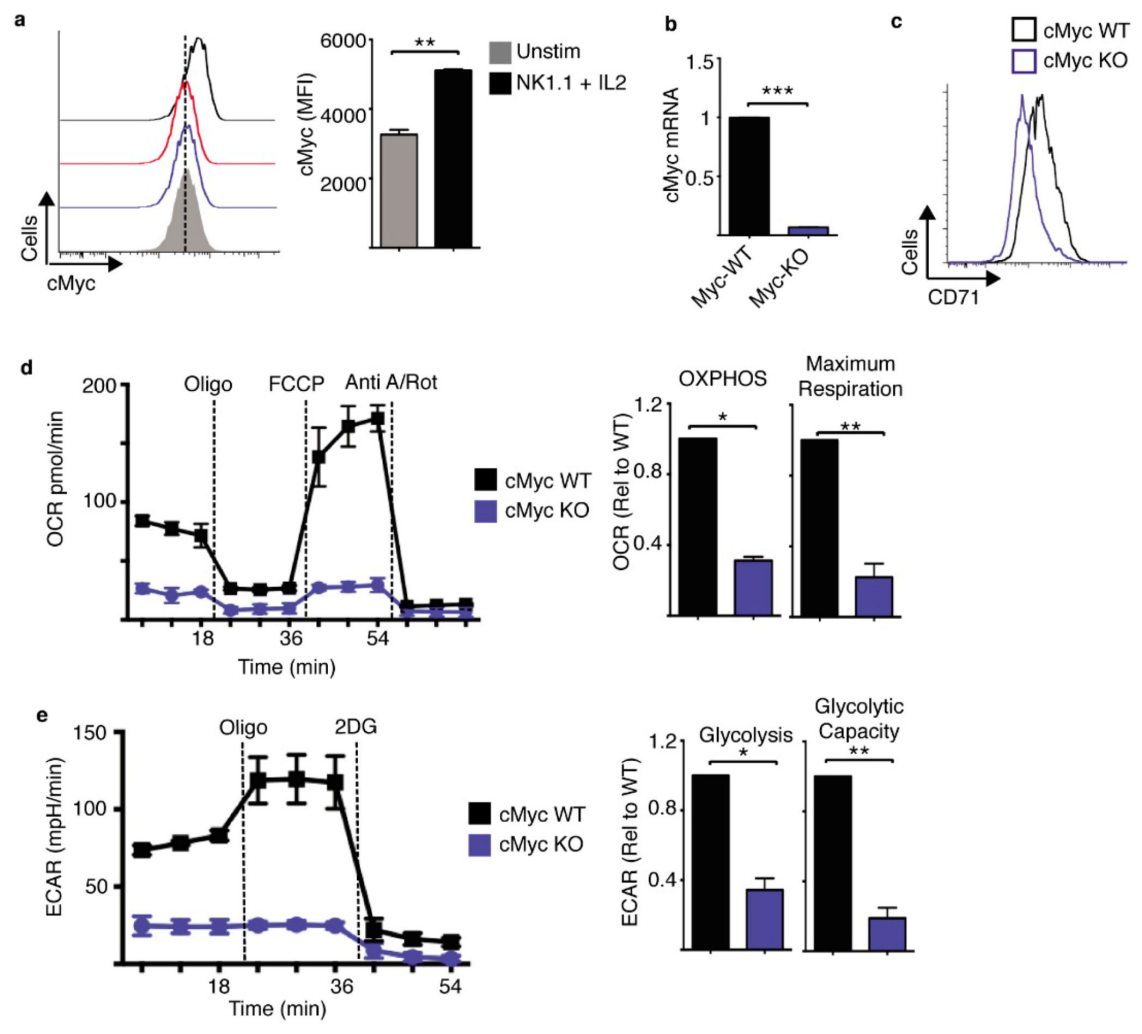
cMyc is required for NK1.1 plus IL2-induced NK cell metabolism. (**a–e**) Cultured (6 days in IL15 10 ng/mL) from cMyc^fl/fl^ Tamox-Cre and cMyc^wt/wt^ Tamox-Cre were treated with tamoxifen (0.6 μM) and were purified and stimulated with a plate bound α-NK1.1 antibody (10 μg/mL) plus IL2 (20 ng/mL) for 18 h. Cells were analysed by flow cytometry for cMyc and CD71 expression (**a,c**) or by quantitative rtPCR for cMyc mRNA expression (**b**). Alternatively, rates of OXPHOS (**d**) or glycolysis (**e**) were measured using the seahorse extracellular flux analyser. Data is representative (**a,c,d,e**) or mean ± SEM (**a,b,d,e**) of 3 independent experiments. Data was analyzed using a students *t*-test (a) or a one sample *t*-test against a theoretical value of 1 (**b,d,e**) (* *p* < 0.05, ** *p* < 0.01).

NK1.1 plus IL2 stimulated NK cells have elevated mTORC1 signalling relative to NK cells stimulated with NK1.1 or IL2 alone (Figure 5a,b). mTORC1 signalling was measured by immunoblot analysis of the phosphorylation of the mTORC1 substrate p70 S6-kinae (S6K) on threonine 389 and serine 421/4 and the downstream S6K target S6 ribosomal protein (S6) on serine 235/6 (Figure 5a). Similar changes in S6 phosphorylation were observed using flow cytometry (Figure 5b). mTORC1 signalling was required for elevated NK cell metabolism, as NK cells showed impaired metabolism in response to NK1.1 plus IL2 when mTORC1 was inhibited using rapamycin. Rapamycin treated NK cells had reduced rates of glycolysis and OXPHOS and reduced expression of nutrient receptors such as CD71 and CD98 (Figure 5c–e, Supplementary Figure S5). cMyc protein expression was also measured by flow cytometry and found to be highest in NK1.1 plus IL2 stimulated NK cells when compared to those stimulated with IL2 or NK1.1 alone (Figure 6a). Therefore, we deleted the cMyc gene in NK cells using cMyc^flox^ Tamox-Cre mice (Myc-KO), as described previously [7]. This transgenic system efficiently deletes cMyc as confirmed by the loss of cMyc mRNA and CD71 expression, a cMyc target gene (Figure 6b,c, Supplementary Figure S5). cMyc-KO NK cells had substantially reduced rates of glycolysis and OXPHOS when stimulated with NK1.1 plus IL2 (Figure 6d,e).

**Figure 7:**
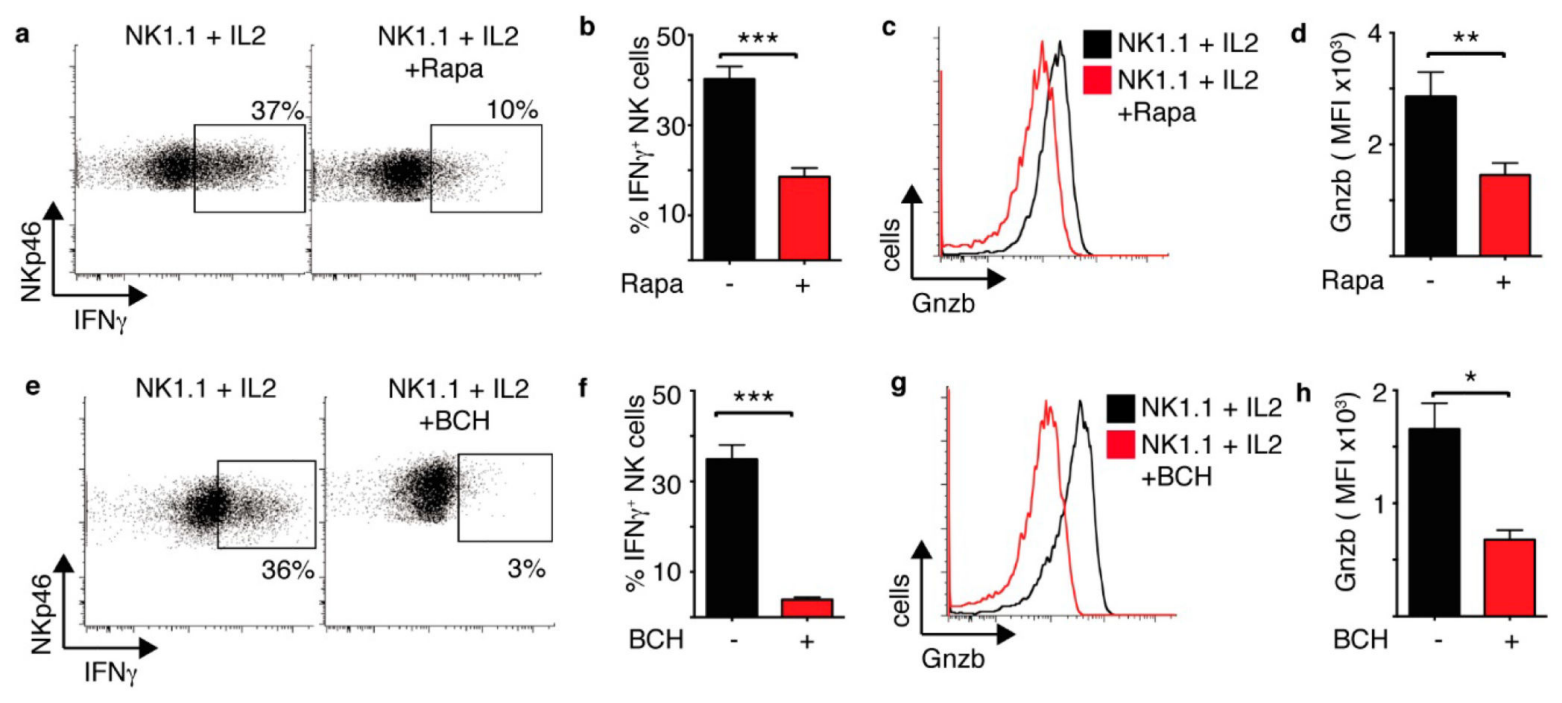
mTORC1 and cMyc are required for NK1.1 plus IL2-induced NK cell effector functions. (**a–h**) Cultured NK cells (6 days in IL15 10 ng/mL) were stimulated with a plate bound α-NK1.1 antibody (10 μg/mL) plus IL2 (20 ng/mL) ± rapamycin (20 nM) (**a**,**d**) or ±BCH (25 mM) (**e–h**) as indicated. Cells were analysed by flow cytometry for the production of IFNγ (**a,b,e,f**) or granzyme B expression (**c,d,g,h**). Data is representative (**a,c,e,g**) or mean ± SEM (**b,d,f,h**) of 3–6 independent experiments. Data was analyzed using a paired students *t-*test (* *p* < 0.05, ** *p* < 0.01, *** *p* < 0.001).

mTORC1 and cMyc-controlled metabolism is important for NK1.1 plus IL2 induced NK cell effector function. Rapamcyin treatment resulted in the inhibition of IFNγ production and granzyme B expression in NK1.1 plus IL2 stimulated NK cells (Figure 7a–d). Amino acid uptake through the system L amino acid transporter Slc7a5 is required to sustain mTORC1 activity and cMyc expression [7]. An inhibitor of Slc7a5 mediated amino acid transport, 2-amino-2-norbornanecarboxylic acid (BCH) also inhibited NK1.1 plus IL2 induced IFNγ production and granzyme B expression. (Figure 7e–h).

### mTORC1 and cMyc-Dependent Metabolism Sustains the Function and Viability of Tumour Interacting NK Cells

**Figure 8:**
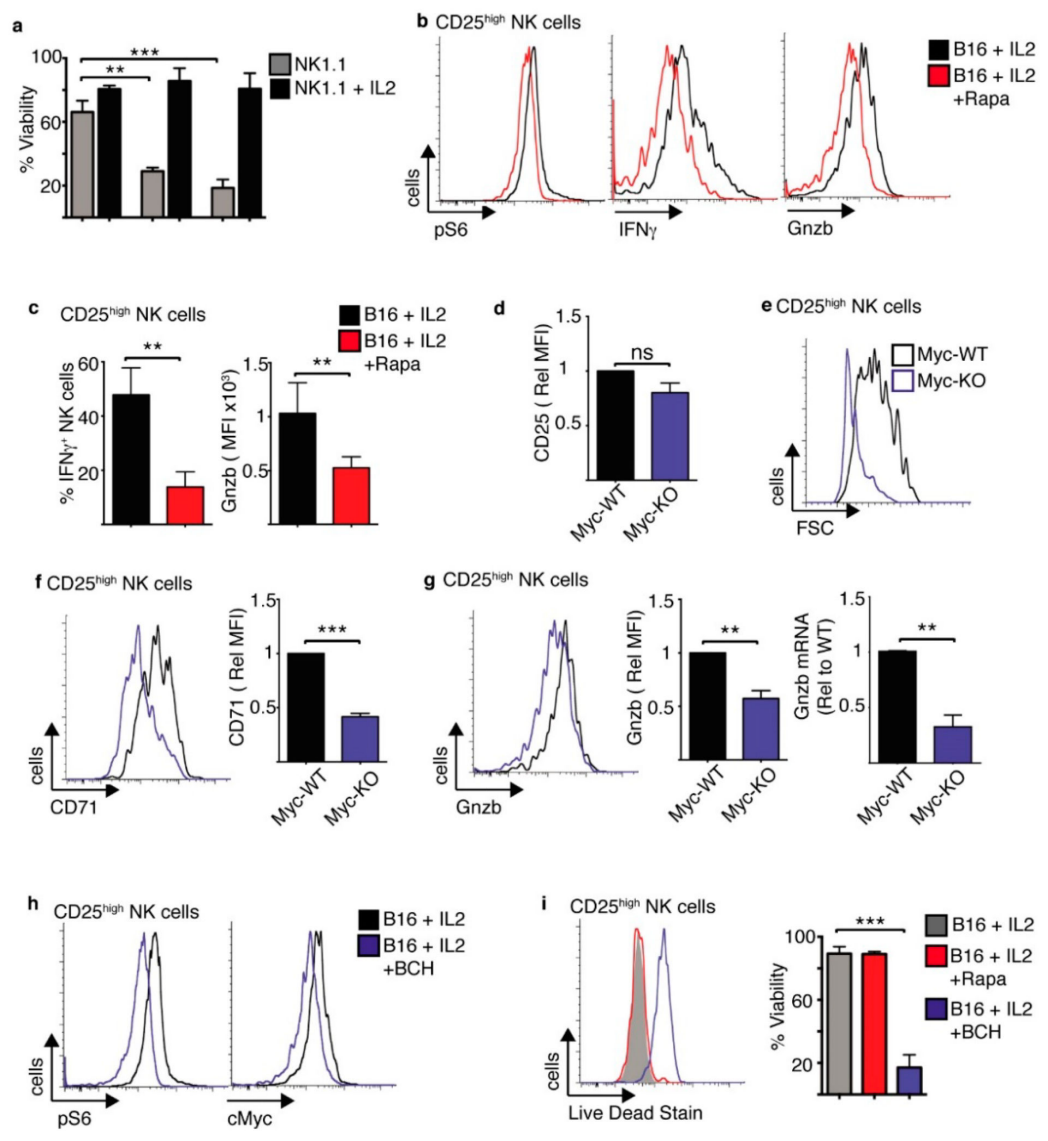
mTORC1/cMyc promote the function and survival of tumour interacting CD25^high^ NK cells. **(a)** Cultured NK cells (3 days in IL15 10 ng/mL) were purified and stimulated with a plate bound α-NK1.1 antibody (10 μg/mL) ± IL2 (20 ng/mL) for 24–72 h and analysed by flow cytometry for cell viability. (**b–c**) Cultured NK cells (6 days in IL15 10 ng/mL) were purified and then co-cultured with B16 melanoma cells or for 18 h, washed and put back into culture with IL2 (20 ng/mL) ± rapamycin for 24 h before analysis of CD25^high^ NK cells by flow cytometry for phosphorylated S6 ribosomal protein (pS6), IFNγ production and granzyme B expression. (**d–g**) Cultured (6 days in IL15 10 ng/mL) from cMyc^fl/fl^ Tamox-Cre and cMyc^wt/wt^ Tamox-Cre were treated with tamoxifen (0.6 μM) and then co-cultured with B16 melanoma cells with IL2 (20 ng/mL) for 18 h before analysis by flow cytometry for cell size and the expression of CD25, cell size, CD71, granzyme B and granzyme B mRNA by rtPCR (**g**). (**h,i**) Cultured NK cells (6 days in IL15 10 ng/mL) were purified and then co-cultured with B16 melanoma cells or for 18 h, washed and put back into culture with IL2 (20 ng/mL) ± BCH (25 mM), ± rapamycin (20 nM) as indicated for 24 h before analysis of CD25^high^ NK cells by flow cytometry for phosphorylated S6 ribosomal protein (pS6), cMyc and cell viability. Data is representative (**b,e,f,g,h,i**) or mean ± SEM (**a,c,d,f,g,i**) of 3–5 independent experiments. Data was analyzed using a one way ANOVA with a tukey post test or a paired students *t*-test. (** *p* < 0.01, *** *p* < 0.001).

The data show that NK1.1 stimulated NK cells induce modest changes in cellular metabolism over the course of 18 h. However, these cells do not survive for prolonged periods and the majority of NK cells are dead after 48 h stimulation (Figure 8a). Indeed, NK1.1 stimulated NK cells have been described to undergo activation induced cell death previously [21]. However, the addition of IL2 not only enhances NK cell metabolism and function, it also promotes cell survival allowing NK1.1-stimulated NK cells to function for extended durations (Figure 8a). Therefore, we next considered whether IL2-induced metabolism is important for the effector function and survival of NK cells interacting with tumour cells. NK cells were co-cultured with B16 melanoma cells for 18 h and then with IL2 in the presence or absence of rapamycin for a further 18 h. As expected, rapamycin treatment inhibited mTORC1 signalling in the CD25^high^ NK cells, as shown by the reduction in pS6 levels (Figure 8b). This led to reduced IFNγ production and granzyme B expression compared to untreated controls (Figure 8b,c). Myc-KO and Myc-WT NK cells were cultured with B16 melanoma cells for 18 h in the presence of IL2. While CD25 expression was induced in both Myc-KO and Myc-WT NK cells, CD25^high^ Myc-KO NK cells were substantially smaller in size (Figure 8d,e). In addition, CD25^high^ Myc-KO NK cells had reduced CD71 and granzyme B expression (Figure 8f,g). Experiments were also carried out using BCH, which inhibits Slc7a5-mediated amino acid transport. BCH treated NK cells had reduced levels of both mTORC1 signalling and reduced cMyc protein expression (Figure 8h, Supplementary Figure S6a), which was associated with reduced IFNγ production and granzyme B expression (Supplementary Figure S6b,c). While inhibition of mTORC1 alone with rapamycin had no effect on NK cell viability, inhibition of both mTORC1 and cMyc signalling following BCH treatment resulted in a dramatic decrease in NK cell viability (Figure 8i).

Taken together, the data show that activating receptor mediated stimulation of NK cells leads to the induction of CD25 expression, facilitating IL2-dependent increases in NK cell metabolism that are required for robust NK cell effector function and prolonged NK cell survival.

## Discussion

As part of the innate immune system NK cells are primed for immediate cytokine production, such as IFNγ, and one NK cell can kill multiple targets before eventually becoming anergic or undergoing apoptosis [22–24]. However, it is now clear that NK cells can also have long-term roles and can function alongside the adaptive immune response [25–27]. While cytokine-induced activation has been shown to facilitate prolonged NK cells responses [1,2,5], less is known about the role of activating NK cell receptors such as NK1.1 in bridging innate and adaptive immunity.

This study reveals the co-operation of receptor ligation and IL2 cytokine signalling in terms of changes in NK cell metabolism. This data provides mechanistic insight into previous studies that explored receptor and cytokine stimulated NK cell responses. For instance, IL2 was shown to be necessary for NK1.1 induced proliferation and IL2-activated NK cells stimulated through NK1.1 had increased cytotoxicity toward tumour target cells [28,29]. Another study showed that increased IL2 availability following the depletion of Tregs led to increased NK cell dependent tumour cell killing [30]. However, the mechanisms underpinning this co-operation between receptor ligation and IL2 were not clear. This study demonstrates for the first time that NK cells stimulated either via NK1.1 receptor ligation or through tumour interactions express high levels of CD25, the high affinity IL2 receptor subunit, and provides evidence that this makes NK cells more responsive to IL2 cytokine. Indeed, the co-operation of IL2 with other cytokines has previously been shown to be due to the ability of cytokines such as IL12, IL18 and IL15 to induce CD25 expression on NK cells [1,2,5]. A recent study has also shown that ligation of the Fc receptor CD16 on NK cells can also promote the expression of CD25 [31]. Our data now show that the key activating NK cell receptor NK1.1 also induces the expression of CD25 on NK cells. Taken together, this argues that the induction of CD25 on NK cells is a common mechanism to increase the sensitivity of NK cells to the adaptive cytokine IL2.

It is interesting that only a proportion of NK cells up-regulated CD25 when co-cultured with tumour B16 melanoma cells, which is in contrast to uniform upregulation of this receptor in NK cells activated through the NK1.1 receptor. Increasing the ratio of tumour cells to NK cells resulted in increased frequency of CD25 expression on NK cells. These data suggest that the activating ligands may be limiting on these melanoma tumour cells. In addition, NK cells co-cultured with different tumour cells showed different patterns of CD25 expression, highlighting the variability amongst cancers and their interactions with NK cells. The data clearly showed that CD25^high^ NK cells have heightened metabolic and functional responses to IL2 produced by local T cells compared to CD25_low_ counterparts in the same co-culture. This IL2 cytokine promotes signalling through mTORC1 and cMyc pathways in these CD25^high^ NK cells. In activated T cells, it has been also been shown that elevated cMyc signalling is a feature of CD25^high^ expressing T cells [32]. Similarly, studies in human NK cells have shown that CD25 expression correlates with target cell-induced cytokine secretion, highlighting an important role for IL2 signalling [33]. There has been substantial interest in exploring the use of IL2 in cancer immunotherapies, which have been hampered by side effects including the expansion of Tregs [34–36]. More recently, efforts have been made to generate mutant forms of IL2 like “super-2” and IL2Cx that make the IL2 cytokine CD25-independent, thus avoiding the unwanted expansion of Tregs [35,37,38]. These mutant IL2 cytokines may also provide beneficial effects for NK cell responses as they would be predicted to promote robust metabolic and functional responses in all NK cells whether CD25^high^ or CD25^low^.

Numerous studies now support a key role for mTORC1 and cMyc signalling, activated by IL2 and IL15 cytokines, in shaping NK cell metabolism to support NK cell effector function [5,7,19,39,40]. However, other signalling pathways may contribute in response to other cytokines; recently IL18 was shown to promote mTORC1-independent metabolic and functional responses in murine NK cells [41]. Meanwhile, other cyokines, such as IL12 support the metabolic actions of IL2 through the induction of CD25 expression [1,5,7]. In this study, we now show that the ligation of activating receptors also supports IL2-mediated reponses in NK cells through inducing CD25 expression. In this context, IL2 is also critical in supporting NK cell longevity by counteracting activation-induced cell death. It is well established that the ligation of NK cell activating receptors can cause activation-induced cell death [21,23,24,42]. At the same time, IL2 is known to increase NK cell longevity and enhance serial killing by NK cells and this involves the induction of the anti-apoptotic protein Bcl2 through the activation of STAT5 [39,43–45]. Herein, we show that IL2-induced metabolism through mTORC1 and cMyc are important for the survival of receptor activated NK cells, leading to prolonged NK cell responses. In addition to functional exhaustion, NK cells from solid tumours have been demonstrated to display poor persistence and short life span [46,47]. There are multiple conditions within the tumour microenvironment that will contribute to this NK cell dysfunction, including low concentrations of glucose and also low levels of IL2. As tumour infiltrating T cells are often anergic and express immune-checkpoints such as PD-1 and CTLA-4 [48], there will be a paucity of T cell-derived IL2 that will impact upon tumour infiltrating NK cells. There is now evidence that NK cells contribute to the therapeutic effect of checkpoint inhibitor therapies as some tumour infiltrating NK cells can express PD-1, but it is tempting to speculate that increased T cell production of IL2 within the tumour also makes an important contribution to this effect [49]. Taken together, the data suggests that the co-operation of signals from activating receptors and IL2 are crucial for modulating NK cell metabolism to facilitate the induction of robust and prolonged anti-tumour NK cell responses.

The advent of new “off-the-shelf” NK cell based cancer immunotherapies opens new possibilities for the manipulation of NK cells so that they are better equipped in a solid tumour microenvironment. Technologies like production of CAR-NK cells, NK92-CAR-NK and induced pluripotent stem cells (iPSC) make it possible to over-express molecules that could enhance NK cell metabolic responses in a nutrient deprived area [50]. Indeed, the greatest hurdle in treating solid cancer remains overcoming the hostile conditions within the tumour microenvironments. Understanding NK cell metabolism in greater detail will pave the way towards strategies that target metabolic signalling and pathways for improved immunotherapies, using the “natural killers” to their full potential.

## Materials and Methods

### Mice

C57BL/6J mice were puchased from Harlan (Bicester, UK) or were bred in-house. Mice with loxP sites inserted flanking exon 2 of the *Myc* gene (B6.129S6-Myc^tm2Fwa^/Mmjax), were from The Jackson Laboratory. Transgenic mice expressing a tamoxifen inducible cre-recombinase (Gt(ROSA)26Sor < tm2(cre/ERT2) Brn/Cnrm) were obtained from the European Mouse Mutant Archive (EMMA). All mice were bred and maintained in compliance with EU and the Health Products Regulatory Authority regulations with the approval of the University of Dublin’s ethical review board (AE19136/P033 awarded 02/04/2015).

### Cell Culture

Cells were cultured in RPMI media +l-glutamine (Invitrogen, California, USA) supplemented with 10% heat-inactivated FCS (Labtech International, East Sussex, UK) 50 μM β-Mercaptoethanol (Sigma, Arklow, Ireland), 1% penicillin/streptomycin (Invitrogen, Waltham, California, USA/Biosciences, Dublin, Ireland). For NK cell culture, splenocytes were isolated from the murine spleen and cultured in IL-15 (10 ng/mL or 12.5 ng/mL, Peprotech, London, UK or NIH, USA; in RPMI media, Biosciences, Dublin, Ireland) for 4 days. On day 4, the cells were supplemented with IL-15 (10 ng/mL or 12.5 ng/mL Peprotech/NIH) and cultured for a further 2 days. Where indicated, splenocytes isolated from *cMyc^−/−^* (cMyc^*flox/flox*^ xTamox-cre) or WT (cMyc^WT/WT^ × Tamox-cre) mice were cultured for 4 days in IL-15 (10–12.5 ng/mL, Peprotech, London, UK/NIH, USA; in RPMI media, Biosciences, Dublin, Ireland) in the presence of 4-hydroxytamoxifen (0.6 μM, Sigma, Arklow, Ireland) to induce cre recombinase mediated excision of the floxed cMyc exon. 4-Hydroxytamoxifen (0.6 μM, Sigma, Arklow, Ireland) was re-added on day 4 when cultures were fed with IL-15 (10 or 12.5 ng/mL, Peprotech, London, UK/NIH, USA).

On day 6, cells were stimulated for 18 h with αNK1.1 plate-bound cross-linking antibody (10 μg/mL, PK136, BD Biosciences, SanDiego, USA) in the presence of either IL15 (5–7.5 ng/mL) or IL2 (20 ng/mL, NCI preclinical repository); or IL2 (20 ng/mL) alone; or IL2 (20 ng/mL) and IL12 (10 ng/mL, Miltenyi Biotech, Bergisch Gladbach, Germany). Unstimulated controls were supplemented with low dose IL15 (5–7.5 ng/mL). For B16 co-culture experiments, 6 day cultured NK cells were purified and co-cultured for 18 h with or without B16 melanoma cells in the E:T ratio of 2:1 supplemented with low dose IL15 (7.5 ng/mL) and were cultured for a further 18 h in media containing IL15 (7.5 ng/mL) or IL-2 (20 ng/mL) in the presence or absence of Rapamycin (20 nM, Fisher, Hampton, New Hampshire, USA) or BCH (25 mM, Sigma, Arklow, Ireland). For B16:NK:T co-cultures, 6 day cultured NK cells were purified and co-cultured with or without B16 melanoma cells in the E:T ratio of 1:2 supplemented with low dose IL15 (7.5 ng/mL). Naïve T cells or T cells that were activated for 24 h were then added to the NK:B16 co-cultures in an NK:T ratio of 1:1. The co-cultures were allowed to incubate for 18 h prior to FACS analysis. For T cell activation, lymph nodes were extracted from B6 mice and were processed and purified with α-CD3 purification kit (Mojosort, Biolegend, San Diego, USA) T cells were then cultured at a concentration of 5 × 10^6^ in IL7 (5 ng/mL, Naïve, R&D, Abingdon, UK) or activated with α-CD3 (1 μg/mL, BD, San Diego, USA) and α-CD28 (2 μg/mL, BD, San Diego, USA) for 24 h before being co-cultured with NK cells and B16 cells. In Myc KO co-culture experiments, 6 day cultured Myc KO and WT NK cells were co-cultured with B16 melanoma cells in the presence of IL2 in the E:T ratio of 1:1. Where indicated, NK cells were purified by magnetic-activated cell sorting (MACS) using the NK cell isolation kit or the EasySep™ Mouse NK cell isolation kit (Miltenyi Biotech, Bergisch Gladbach, Germany and Stemcell technologies Vancouver, Canada, respectively) from the culture on day 6.

For B16 co-culture experiments, 6 day cultured NK cells were co-cultured for 18 h with or without B16 melanoma cells in the E:T ratio of 2:1 supplemented with low dose IL15(7.5 ng/mL) and were cultured for a further 18 h in media containing IL2 (20 ng/mL) or IL15 (7.5 ng/mL).

B16, YAC-1, RMA and RMA-S cell lines were purchased from the American Type Culture Collection (Manassas, Virginia, USA).

B16 melanoma cells, CT26 and Lewis lung carcinoma cells were grown in DMEM GlutaMax (Biosciences, Dublin, Ireland) medium supplemented with 10% FCS (Labtech International, East Sussex, UK) and 1%Penicillin/Streptomycin (Biosciences, Dublin, Ireland). The cells were passaged when they reached 80% confluency.

YAC-1 RMA and RMA-S cell were cultured in RPMI medium (Biosciences, Dublin, Ireland) containing L-glutamine, supplemented with 10% FCS (Labtech International, East Sussex, UK) and 1% Penicillin/Streptomycin (Biosciences, Dublin, Ireland) The cells were cultured at 0.2–0.4 × 10^6^/mL and were passaged every 2 days before they reached a concentration of 3 × 10^6^/mL.

For glycolytic inhibition, 6 day cultured NK cells (or 3 day cultured NK cells where indicated) were stimulated with α-NK1.1 cross-linking antibody (10 μg/mL) plus IL2 (20 ng/mL) in the presence or absence of 2-deoxyglucose, 2DG (1 mM, Sigma, Arklow, Ireland) or Oxalate (2 mM, Sigma, Arklow, Ireland) for 18 h. For OXPHOS inhibition, 6 day cultured NK cells were stimulated with α-NK1.1 cross-linking antibody (10 μg/mL, BD, San Diego, USA) plus IL2 (20 ng/mL) in the presence or absence of Oligomycin (4 nM, Sigma, Arklow, Ireland) for 18 h. For mTORC1 inhibition, 6 day cultured NK cells were stimulated with αNK1.1 cross-linking antibody (10 μg/mL) plus IL2 (20 ng/mL) in the presence or absence of Rapamycin (20 nM, Fisher, Hampton, New Hampshire, USA) for 18 h. Functional responses were measured by flow cytometry following stimulation. For SLC7A5 inhibition experiments, the concentration of amino acids in RPMI was diluted two fold using HBSS (Invitrogen, Waltham, California, USA) in the presence or absence of 2-amino-2-norbornanecarboxylic acid (BCH, 25 mM Sigma, Arklow, Ireland).

### Flow Cytometry

Cells were incubated for 10 min at 4 °C with Fc blocking antibody CD16/CD32 (2.4G2) and subsequently stained for 20 min at 4 °C with saturating concentrations of fluorphore conjugated antibodies. Antibodies used were as follows: NK1.1-eFluor 450 (PK136), NK1.1-BV421 (PK136), NKp46-PerCP eFluor 710 (29A1.4), CD3-FITC (145-2C11), TCRβ-APC (H57-597), CD25-APC-Cy7 (PC61), CD71-PE (C2), CD71-BV510 (C2) IFNγ-APC (XMG1.2), Granzyme B-PE-Cy7 (NGZB), CD98-PE (RL388), pS6(D57.2.2E), cMyc-PE (D84C12), purchased from eBiosciences, San Diego, California, USA, Cell signaling, Danvers, Massachusetts, USA, Biolegend, SanDiego, USA and BD Biosciences, San Diego, USA. Live lymphocytes were gated according to their forward scatter (FSC-A) and side scatter; single cells according to their FSC-W and FSC-A, NK cells were identified as NK1.1^+^, NKp46^+^ and CD3^−^ or TCRβ^−^. For intracellular staining, the cells were incubated for 4 h with the protein transport inhibitor GolgiPlug™ (BD Biosciences, San Diego, USA). For fixation and permeabilization of the cells, the Cytofix/Cytoperm kit from BD Biosciences was used according to manufacturer’s instructions. Data were acquired on a FACS Canto, (Becton Dickinson, Franklin Lakes, New Jersey, USA) and analysed using FlowJo software (TreeStar, Ashland, USA)

For viability studies, 3 day cultured NK cells were activated with indicated stimuli for 24, 48 and 72 h and stained with Live/dead™ fixable Aqua stain (Invitrogen, Waltham, California, USA) according to the manufacturer’s instruction. Viability was analysed as percentage of NK cells negative for Live/dead™ Aqua stain.

For pS6 staining, stimulated NK cells were fixed with cytofix/cytoperm before being stained for pS6 antibody (D57.2.2E) (unconjugated, Cell signalling, Danvers, Massachusetts, USA). After 30 min incubation, the cells were washed, and stained for extracellular antibodies, along with a secondary antibody for pS6 (PE, donkey anti-rabbit, Jackson Immunoresearch, Cambridgeshire, UK).

For cMyc staining, stimulated NK cells were transferred to 96 well plate, stained for extracellular markers and incubated for 20 min. Following the incubation, the cells were washed with FACS buffer and were incubated in FACS buffer containing 0.2% Tween-20 and 0.5% PFA (Sigma, Arklow, Ireland) for a minimum of 2 h at RT in dark. The cells were thoroughly washed with the standard FACS buffer (20% RPMI in PBS), and were stained for cMyc using PE conjugated cMyc antibody- (D84C12, Cell signalling, Danvers, Massachusetts, USA) for 1 h at RT.

For Transferrin uptake, NK cells were washed in serum free RPMI supplemented with 0.5% BSA, and then incubated in serum free RPMI supplemented with 5% BSA for 1 h at 37 °C. After incubation, cells were then with or without 5 μg/mL of Transferrin conjugated with Alexa Fluor 647 (Invitrogen, Waltham, California, USA) for 10 min at 37 °C or 4 °C. Cells were transferred to ice and washed first with PBS supplemented with 150 mM NaCl and 20 mM citric acid, pH 5 and then with ice cold RPMI + 0.5% BSA before analysis by flow cytometry.

### Seahorse Metabolic Flux Analyzer

For real-time analysis of the extracellular acidification rate (ECAR) and oxygen consumption rate (OCR) of purified and expanded NK cells cultured under various conditions, a Seahorse XF-24 Analyzer, or a Seahorse XFe-96 Analyzer Seahorse (Agilent Technologies, Santa Clara, CA, USA) was used. In brief, 500,000 to 750,000 MACS purified, expanded NK cells were added to a 24-well XF Cell Culture Microplate, 200,000 MACS purified NK cells to a 96-well XFe Cell Culture Microplate (Agilent Technologies, Santa Clara, CA, USA). All cell culture plates were treated with Cell-Tak™ (BD Pharmingen, San Diego, USA) to ensure that the NK cells adhere to the plate. Sequential measurements of ECAR and OCR following addition of the inhibitors (Sigma, Arklow, Ireland) oligomycin (2 μM), FCCP (1 μM), rotenone (100 nM) plus antimycin A (4 μM), and 2-deoxyglucose (2DG, 30 mM) allowed for the calculation of basal glycolysis, glycolytic capacity, basal mitochondrial respiration, and maximal mitochondrial respiration.

### Western Blot Analysis

For western blot analysis, cells were harvested, washed twice with ice-cold PBS and lysed at 1 × 10^7^/mL in lysis buffer containing 50 mM Tris Cl pH 6.7, 2% SDS, 10% glycerol, 0.05% Bromphenol Blue, 1 μM DTT, phosphatase- and protease inhibitors. Samples were denatured at 95 °C for 10 min, separated by SDS-PAGE and transferred to a PVDF membrane. Blots were probed with antibodies recognizing phosphorylated-S6^S235/6^ (D57.2.2E), phospho-S6K^T389^ (108D2), phosphor-S6K^T421/S424^, (Cell Signaling Technologies, Danvers, Massachusetts, USA) S6(C-8), S6K(C-18) (Santa Cruz Biotechnology, Dallas, Texas, USA).

### Quantitative Real Time PCR

Cultured NK cells were purified using MACS purification EasySep™ Mouse NK cell isolation kit (Stemcell technologies, Vancouver, Canada), prior to stimulation. RNA was isolated using the RNeasy RNA purification mini kit (QIAGEN, Hilden, Germany), according to manufacturer’s protocol. From purified RNA, cDNA was synthesized using the reverse-transcriptase kit qScript cDNA synthesis kit (Quanta Biosciences, Beverly, MA, USA). Real time PCR was performed in triplicates in a 96-well plate using iQ SYBR Green-based detection on an ABI 7900HT fast qPCR machine. For the analysis of mRNA levels the derived values were normalized to the average of values obtained from the mRNA analysis of 3 different house-keeping genes- RPLP0, GAPDH, and HPRT.

### Primers

*Rplp0* forward: 5′-CATGTCGCTCCGAGGGAAG-3′

*Rplp0* reverse: 5′-CAGCAGCTGGCACCTTATTG-3′

*cMyc* forward: 5′-GCGTTGGAAACCCCGACAG-3′

*cMyc* reverse: 5′-CTTCCAGATATCCTCACTGGGC-3′

*Gapdh* forward: 5′-CATGGCCTTCCGTGTTCCTA-3′

*Gapdh* reverse: 5′-CCTGCTTCACCACCTTCTTGAT-3′

*Hprt* forward: 5′-TGATCAGTCAACGGGGGACA-3′

*Hprt* reverse: 5′-TTCGAGAGGTCCTTTTCACCA-3′

### Statistical Analysis

GraphPad Prism 6.00 (GraphPad Software, www.graphpad.com) was used for statistical analysis. A one-way ANOVA with the Tukey post hoc test was used for multiple comparisons. Students *t*-test was used when there were only 2 data sets for comparison. For comparison of relative values, a one-sample *t*-test was usesd to calculate *P* values with the theoretical mean set to 1.00. A *p*-value < 0.05 was considered as statistically significant.

## Supplementary Material

The supplementary materials are available online at https://doi.org/10.20900/immunometab20190014.

Supplementary Materials

## References

[1] Lee SH, Fragoso MF, Biron CA (2012). Cutting edge: a novel mechanism bridging innate and adaptive immunity: IL-12 induction of CD25 to form high-affinity IL-2 receptors on NK cells. J Immunol.

[2] Leong JW, Chase JM, Romee R, Schneider SE, Sullivan RP, Cooper MA (2014). Preactivation with IL-12, IL-15, and IL-18 induces CD25 and a functional high-affinity IL-2 receptor on human cytokine-induced memory-like natural killer cells. Biol Blood Marrow Transplant.

[3] Fehniger TA, Cooper MA, Nuovo GJ, Cella M, Facchetti F, Colonna M (2003). CD56bright natural killer cells are present in human lymph nodes and are activated by T cell-derived IL-2: a potential new link between adaptive and innate immunity. Blood.

[4] Assmann N, O’Brien KL, Donnelly RP, Dyck L, Zaiatz-Bittencourt V, Loftus RM (2017). Srebp-controlled glucose metabolism is essential for NK cell functional responses. Nat Immunol.

[5] Donnelly RP, Loftus RM, Keating SE, Liou KT, Biron CA, Gardiner CM (2014). mTORC1-dependent metabolic reprogramming is a prerequisite for NK cell effector function. J Immunol.

[6] Keating SE, Zaiatz-Bittencourt V, Loftus RM, Keane C, Brennan K, Finlay DK (2016). Metabolic Reprogramming Supports IFN-gamma Production by CD56bright NK Cells. J Immunol.

[7] Loftus RM, Assmann N, Kedia-Mehta N, O’Brien KL, Garcia A, Gillespie C (2018). Amino acid-dependent cMyc expression is essential for NK cell metabolic and functional responses in mice. Nat Commun.

[8] Viel S, Marcais A, Guimaraes FS, Loftus R, Rabilloud J, Grau M (2016). TGF-beta inhibits the activation and functions of NK cells by repressing the mTOR pathway. Sci Signal.

[9] Zaiatz-Bittencourt V, Finlay DK, Gardiner CM (2018). Canonical TGF-beta Signaling Pathway Represses Human NK Cell Metabolism. J Immunol.

[10] Michelet X, Dyck L, Hogan A, Loftus RM, Duquette D, Wei K (2018). Metabolic reprogramming of natural killer cells in obesity limits antitumor responses. Nat Immunol.

[11] Tobin LM, Mavinkurve M, Carolan E, Kinlen D, O'Brien EC, Little MA (2017). NK cells in childhood obesity are activated, metabolically stressed, and functionally deficient. JCI Insight.

[12] Cong J, Wang X, Zheng X, Wang D, Fu B, Sun R (2018). Dysfunction of Natural Killer Cells by FBP1-Induced Inhibition of Glycolysis during Lung Cancer Progression. Cell Metab.

[13] Keppel MP, Saucier N, Mah AY, Vogel TP, Cooper MA (2015). Activation-specific metabolic requirements for NK Cell IFN-gamma production. J Immunol.

[14] Kelly JM, Darcy PK, Markby JL, Godfrey DI, Takeda K, Yagita H (2002). Induction of tumor-specific T cell memory by NK cell-mediated tumor rejection. Nat Immunol.

[15] Fan Z, Yu P, Wang Y, Fu ML, Liu W, Sun Y (2006). NK-cell activation by LIGHT triggers tumor-specific CD8^+^ T-cell immunity to reject established tumors. Blood.

[16] Lucas M, Schachterle W, Oberle K, Aichele P, Diefenbach A (2007). Dendritic cells prime natural killer cells by trans-presenting interleukin 15. Immunity.

[17] Dubois S, Mariner J, Waldmann TA, Tagaya Y (2002). IL-15Ralpha recycles and presents IL-15 In trans to neighboring cells. Immunity.

[18] Koka R, Burkett P, Chien M, Chai S, Boone DL, Ma A (2004). Cutting edge: murine dendritic cells require IL-15R alpha to prime NK cells. J Immunol.

[19] Marcais A, Cherfils-Vicini J, Viant C, Degouve S, Viel S, Fenis A (2014). The metabolic checkpoint kinase mTOR is essential for IL-15 signaling during the development and activation of NK cells. Nat Immunol.

[20] Arase H, Arase N, Saito T (1996). Interferon gamma production by natural killer (NK) cells and NK1.1+ T cells upon NKR-P1 cross-linking. J Exp Med.

[21] Asea A, Stein-Streilein J (1998). Signalling through NK1.1 triggers NK cells to die but induces NK T cells to produce interleukin-4. Immunology.

[22] Bhat R, Watzl C (2007). Serial killing of tumor cells by human natural killer cells—enhancement by therapeutic antibodies. PLoS One.

[23] Jewett A, Bonavida B (1996). Target-induced inactivation and cell death by apoptosis in a subset of human NK cells. J Immunol.

[24] Jewett A, Bonavida B (1995). Target-induced anergy of natural killer cytotoxic function is restricted to the NK-target conjugate subset. Cell Immunol.

[25] Cooper MA, Colonna M, Yokoyama WM (2009). Hidden talents of natural killers: NK cells in innate and adaptive immunity. EMBO Rep.

[26] Vivier E, Raulet DH, Moretta A, Caligiuri MA, Zitvogel L, Lanier LL (2011). Innate or adaptive immunity? The example of natural killer cells. Science.

[27] Vivier E, Ugolini S, Blaise D, Chabannon C, Brossay L (2012). Targeting natural killer cells and natural killer T cells in cancer. Nat Rev Immunol.

[28] Reichlin A, Yokoyama WM (1998). Natural killer cell proliferation induced by anti-NK1.1 and IL-2. Immunol Cell Biol.

[29] Karlhofer FM, Yokoyama WM (1991). Stimulation of murine natural killer (NK) cells by a monoclonal antibody specific for the NK1.1 antigen. IL-2-activated NK cells possess additional specific stimulation pathways. J Immunol.

[30] Gasteiger G, Hemmers S, Firth MA, Le Floc'h A, Huse M, Sun JC (2013). IL-2-dependent tuning of NK cell sensitivity for target cells is controlled by regulatory T cells. J Exp Med.

[31] Pahl JHW, Koch J, Gotz JJ, Arnold A, Reusch U, Gantke T (2018). CD16A Activation of NK Cells Promotes NK Cell Proliferation and Memory-Like Cytotoxicity against Cancer Cells. Cancer Immunol Res.

[32] Preston GC, Sinclair LV, Kaskar A, Hukelmann JL, Navarro MN, Ferrero I (2015). Single cell tuning of Myc expression by antigen receptor signal strength and interleukin-2 in T lymphocytes. EMBO J.

[33] Rudnicka K, Matusiak A, Chmiela M (2015). CD25 (IL-2R) expression correlates with the target cell induced cytotoxic activity and cytokine secretion in human natural killer cells. Acta Biochim Pol.

[34] Ahmadzadeh M, Rosenberg SA (2006). IL-2 administration increases CD4^+^ CD25(hi) Foxp3+ regulatory T cells in cancer patients. Blood.

[35] Rosenberg SA, Lotze MT, Muul LM, Leitman S, Chang AE, Ettinghausen SE (1985). Observations on the systemic administration of autologous lymphokine-activated killer cells and recombinant interleukin-2 to patients with metastatic cancer. N Engl J Med.

[36] Zhang H, Chua KS, Guimond M, Kapoor V, Brown MV, Fleisher TA (2005). Lymphopenia and interleukin-2 therapy alter homeostasis of CD4^+^CD25^+^ regulatory T cells. Nat Med.

[37] Caudana P, Núñez NG, De La Rochere P, Pinto A, Denizeau J, Alonso R (2019). IL2/Anti-IL2 Complex Combined with CTLA-4, But Not PD-1, Blockade Rescues Antitumor NK Cell Function by Regulatory T-cell Modulation. Cancer Immunol Res.

[38] Porichis F, Hart MG, Massa A, Everett HL, Morou A, Richard J (2018). Immune Checkpoint Blockade Restores HIV-Specific CD4 T Cell Help for NK Cells. J Immunol.

[39] Lord JD, McIntosh BC, Greenberg PD, Nelson BH (2000). The IL-2 receptor promotes lymphocyte proliferation and induction of the c-myc, bcl-2, and bcl-x genes through the trans-activation domain of Stat5. J Immunol.

[40] Marzec M, Liu X, Kasprzycka M, Witkiewicz A, Raghunath PN, El-Salem M (2008). IL-2- and IL-15-induced activation of the rapamycin-sensitive mTORC1 pathway in malignant CD4^+^ T lymphocytes. Blood.

[41] Almutairi SM, Ali AK, He W, Yang DS, Ghorbani P, Wang L (2019). Interleukin-18 up-regulates amino acid transporters and facilitates amino acid-induced mTORC1 activation in natural killer cells. J Biol Chem.

[42] Taga K, Yamauchi A, Kabashima K, Bloom ET, Muller J, Tosato G (1996). Target-induced death by apoptosis in human lymphokine-activated natural killer cells. Blood.

[43] Moriggl R, Topham DJ, Teglund S, Sexl V, McKay C, Wang D (1999). Stat5 is required for IL-2-induced cell cycle progression of peripheral T cells. Immunity.

[44] Lord JD, McIntosh BC, Greenberg PD, Nelson BH (1998). The IL-2 receptor promotes proliferation, bcl-2 and bcl-x induction, but not cell viability through the adapter molecule Shc. J Immunol.

[45] Tsujimoto Y (1998). Role of Bcl-2 family proteins in apoptosis: apoptosomes or mitochondria?. Genes Cells.

[46] Barth MJ, Chu Y, Hanley PJ, Cairo MS (2016). Immunotherapeutic approaches for the treatment of childhood, adolescent and young adult non-Hodgkin lymphoma. Br J Haematol.

[47] Konjevic G, Jurisic V, Jovic V, Vuletic A, Mirjacic Martinovic K, Radenkovic S (2012). Investigation of NK cell function and their modulation in different malignancies. Immunol Res.

[48] Thommen DS, Schumacher TN (2018). T Cell Dysfunction in Cancer. Cancer Cell.

[49] Hsu J, Hodgins JJ, Marathe M, Nicolai CJ, Bourgeois-Daigneault MC, Trevino TN (2018). Contribution of NK cells to immunotherapy mediated by PD-1/PD-L1 blockade. J Clin Invest.

[50] Li Y, Hermanson DL, Moriarity BS, Kaufman DS (2018). Human iPSC-Derived Natural Killer Cells Engineered with Chimeric Antigen Receptors Enhance Anti-tumor Activity. Cell Stem Cell.

