# Supplementary material for: Natural Killer Cells Integrate Signals Received from Tumour Interactions and IL2 to Induce Robust and Prolonged Anti-Tumour and Metabolic Responses
Source: Immunometabolism. Author manuscript; Available in PMC 2019 Oct 8. (PMC6783304; doi:10.20900/immunometab20190014)
Supplement: Supplementary Materials [file EMS84496-supplement-Supplementary_Materials.pdf]

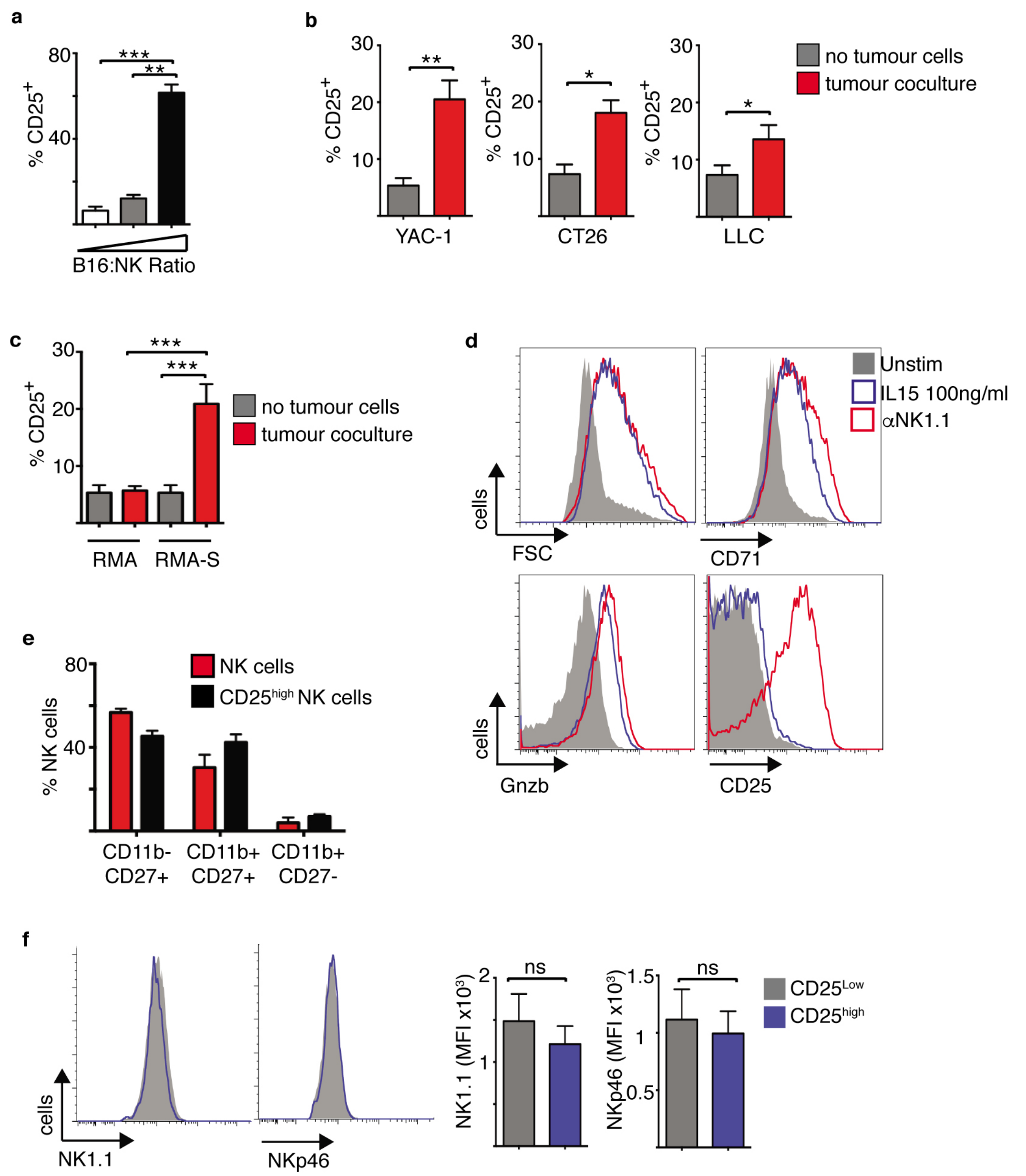

Figure S1. Tumour interactions induce CD25 expression on NK cells. (**a**) Cultured NK cells (6 days in IL15 10 ng/mL) were purified and then co-cultured with or without YAC-1, CT26 and LLC tumour cells at an E:T ratio of 1:4 before analysis by flow cytometry for CD25 expression. (**b**) Cultured NK cells (6 days in IL15 10 ng/mL) were purified and then co-cultured with or without RMA or RMA-S before analysis by flow cytometry for CD25 expression. (**c**) Cultured NK cells (6 days in IL15 10 ng/mL) were left unstimulated or stimulated with plate bound αNK1.1 or high dose IL-15 (100 ng/mL) for 18 h before flow cytometry analysis of cell size (FSC), CD71, Gnzb and CD25 expression. (**d**) Cultured NK cells (6 days in IL15 10 ng/mL) were purified and then cultured ± B16 melanoma cells for 18 h at a E:T ratio of 2:1 for 18 h before analysis by flow cytometry for CD27 and CD11b expression and NK cell subset frequency determined. CD25^{high} NK cells from B16 cocultures (black) were compared to NK cells not cultured with B16 cells (red). (**e**) Cultured NK cells (6 days in IL15 10 ng/mL) were purified and then co-cultured with B16 melanoma cells for 18 h at a E:T ratio of 2:1 for 18 h before analysis by flow cytometry for NK1.1 and NKp46 expression. Data is representative (**c**,**e**) or mean ± SEM (**a**,**b**,**d**,**e**) of 4-5 independent experiments. Data was analyzed using a one way ANOVA with a tukey post test or a paired students *t*-test. (* $p < 0.05$, ** $p < 0.01$, *** $p < 0.001$, ns non-significant).

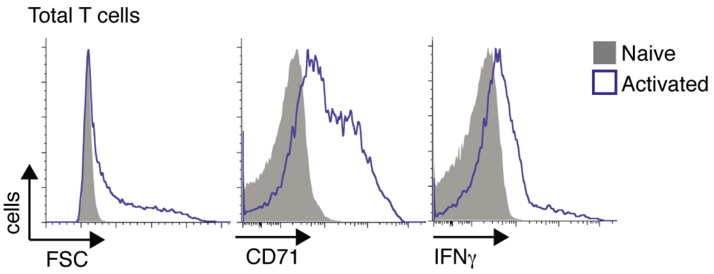

Figure S2. Activation profile of T cells for co-culture experiments. Total T cells were purified from splenocytes and activated with αCD3 (1 µg/mL) and αCD28 (2 µg/mL) for 18 h and analysed for size (FSC), the expression of CD71 and IFN$_\gamma$ production by flow cytometry. Data is representative of 4 independent experiments.

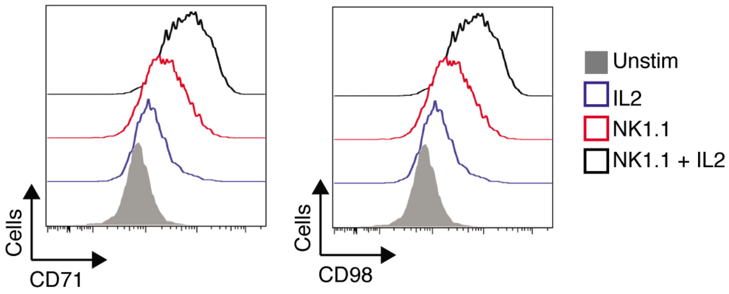

Figure S3. NK1.1 ligation facilitates IL2-mediated CD71 and CD98 expression. Cultured NK cells (6 days in IL15 10 ng/mL) were stimulated with a plate bound α-NK1.1 antibody (10 µg/mL) in media supplemented with low dose IL15 (5 ng/mL) ± IL2 (20 ng/mL) and analysed by flow cytometry for the expression CD71 (left) and CD98 (right). Data is representative of 5 independent experiments.

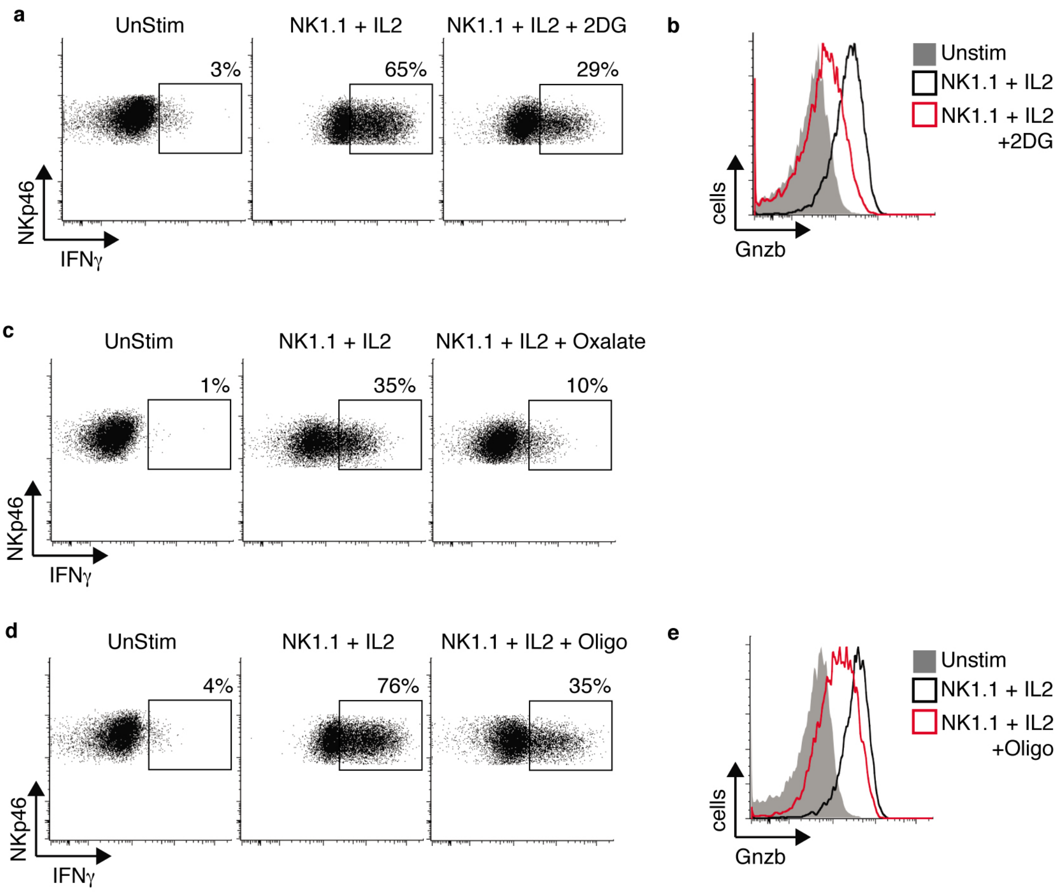

Figure S4. Metabolism is required for NK1.1 plus IL2-induced NK cell effector functions. Cultured NK cells (6 days in IL15 10 ng/mL) were stimulated with a plate bound α-NK1.1 antibody (10 µg/mL) in media supplemented with low dose IL15 (5 ng/mL) + IL2 (20 ng/mL), or left unstimulated (IL15- 5 ng/mL). Inhibitors of glycolysis, 2-deoxyglucose (2DG, 1 mM) (**a**,**b**) or oxalate (2 mM) (**c**), or OXPHOS, oligomycin (4 nM) (**d**,**e**) were added as indicated. Cells were analysed by flow cytometry for the production of IFNγ (**a**,**c**,**d**) and the expression of granzyme B (**b**,**e**). Data is representative of 4 independent experiments.

a

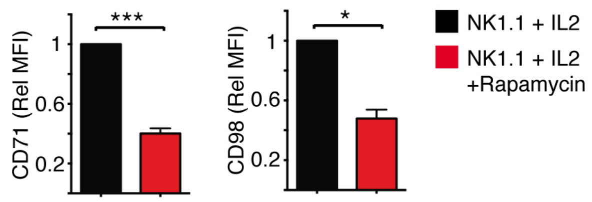

b

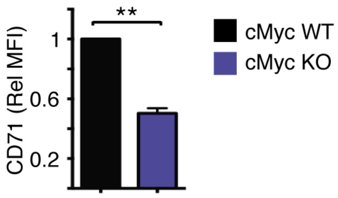

Figure S5. mTORC1 and cMyc nutrient receptor expression in NK1.1 plus IL2-stimulated NK cells. (**a**) Cultured NK cells (6 days in IL15 10 ng/mL) were stimulated with a plate bound α-NK1.1 antibody (10 µg/mL) in media supplemented with low dose IL15 (5 ng/mL) + IL2 (20 ng/mL) ± rapamycin (20 nM), or left unstimulated (IL15- 5 ng/mL). (**b**) Cultured (6 days in IL15 10 ng/mL) from cMyc$^{fl/fl}$ Tamox-Cre and cMyc$^{wt/wt}$ Tamox-Cre were treated with tamoxifen (0.6 µM), purified and stimulated with a plate bound α-NK1.1 antibody (10 µg/mL) plus IL2 (20 ng/mL) for 18 h. Cells were analysed by flow cytometry for the expression of CD71 (**a**,**b**) and CD98 (**a**). Data is mean ± SEM of 3–6 independent experiments. Data was analyzed using a one sample *t*-test against a theoretical value of 1 (* $p < 0.05$, ** $p < 0.01$, ** $p < 0.001$).

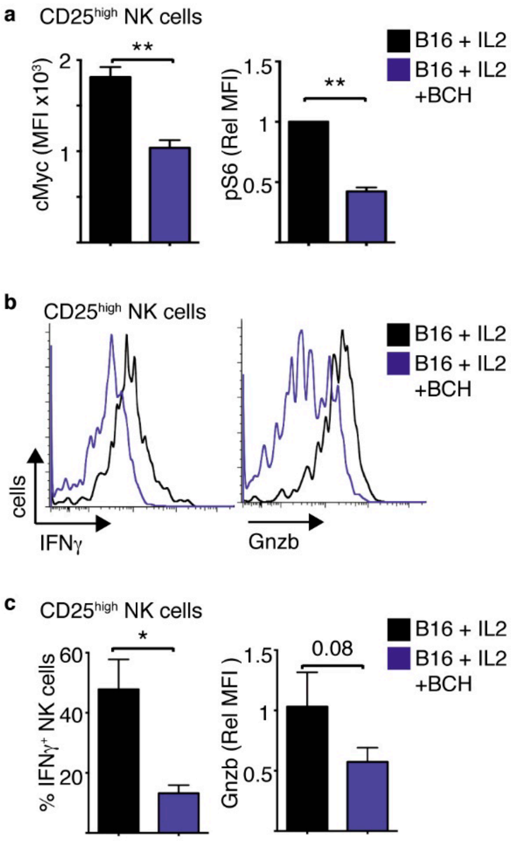

Figure S6. Inhibition of Slc7a5 amino acid uptake disrupts the function of CD25^{high} NK cells. Cultured NK cells (6 days in IL15 10 ng/mL) were purified and then co-cultured with B16 melanoma cells or for 18 h, washed and put back into culture with IL2 (20 ng/mL) ± BCH (25 mM) as indicated for 24 h before analysis of CD25^{high} NK cells by flow cytometry for cMyc and pS6 (a), IFNγ production and granzyme B expression (**b**,**c**). Data is representative (**b**) or mean ± SEM (**a**,**c**) of 3–4 independent experiments. Data was analyzed using a paired students *t*-test or a one sample *t*-test against a theoretical value of 1 (* $p < 0.05$, ** $p < 0.01$).
